# Data on specificity of [^18^F]GE180 uptake for TSPO expression in rodent brain and myocardium

**DOI:** 10.1016/j.dib.2018.04.133

**Published:** 2018-05-05

**Authors:** Maximilian Deussing, Tanja Blume, Lena Vomacka, Christoph Mahler, Carola Focke, Andrei Todica, Marcus Unterrainer, Nathalie L. Albert, Simon Lindner, Barbara von Ungern-Sternberg, Karlheinz Baumann, Andreas Zwergal, Peter Bartenstein, Jochen Herms, Axel Rominger, Matthias Brendel

**Affiliations:** a Dept. of Nuclear Medicine, Ludwig-Maximilians-Universität München, Munich, Germany; b Center for Neuropathology and Prion Research, Ludwig-Maximilians-Universität München, Munich, Germany; c Inst. of Clinical Neuroimmunology, Ludwig-Maximilians-Universität München, Munich, Germany; d Biomedical Center (BMC), Ludwig-Maximilians-Universität München, Munich, Germany; e Roche Pharma Research and Early Development, Neuroscience Discovery, Roche Innovation Center Basel, F. Hoffmann-La Roche Ltd, Basel, Switzerland; f Dept. of Neurology, Ludwig-Maximilians-Universität München, Munich, Germany; g Munich Cluster for Systems Neurology (SyNergy), Munich, Germany; h Department of Translational Brain Research, DZNE - German Center for Neurodegenerative Diseases, Munich, Germany

## Abstract

Data in this article show radioligand uptake (to gamma counter and positron-emission-tomography) as well as polymerase chain reaction analyses of 18 kDa translocator protein (TSPO) quantification. We confirmed specificity of [^18^F]GE180 binding of rodent brain and myocardium by blocking experiments with prior application of non-radioactive GE180, using dynamic in vivo positron-emission-tomography and ex vivo gamma counter measurements. Expression of TSPO was compared between rodent brain and myocardium by quantitative polymerase chain reaction.

**Specifications Table**

**Table:** 

Subject area	*Nuclear Medicine*
More specific subject area	*Preclinical PET imaging of neuroinflammation*
Type of data	*PET images, blocking plots, correlation plots*
How data was acquired	*[*^*18*^*F]GE180 TSPO PET, gamma counter, TSPO qPCR*
Data format	*Quantification of PET and tissue samples, Listmode, NIFTI*
Experimental factors	*C57Bl6 mice, cold GE180, [*^*18*^*F]GE180, Analysis of TSPO expression*
Experimental features	*TSPO PET, TSPO qPCR*
Data source location	*Department of Nuclear Medicine, Munich, Germany*
Data accessibility	*The data is provided within this article*

**Value of the data**

• Blocking by pretreatment with non-radioactive GE180 for quantification of specific [^18^F]GE180 TSPO binding in rodent brain and myocardium to positron-emission-tomography.
• Regional analysis for characterization of [^18^F]GE180 binding specificity in different areas of the rodent brain.
• Data provide information about the physiological relationship between specific 18 kDa translocator protein (TSPO) tracer signal in normal rodent brain and myocardium.
• Quantitative polymerase chain reaction gives additional information about the relative magnitudes of TSPO expression in brain and myocardium.

## 1 Data

**Fig. 1:**
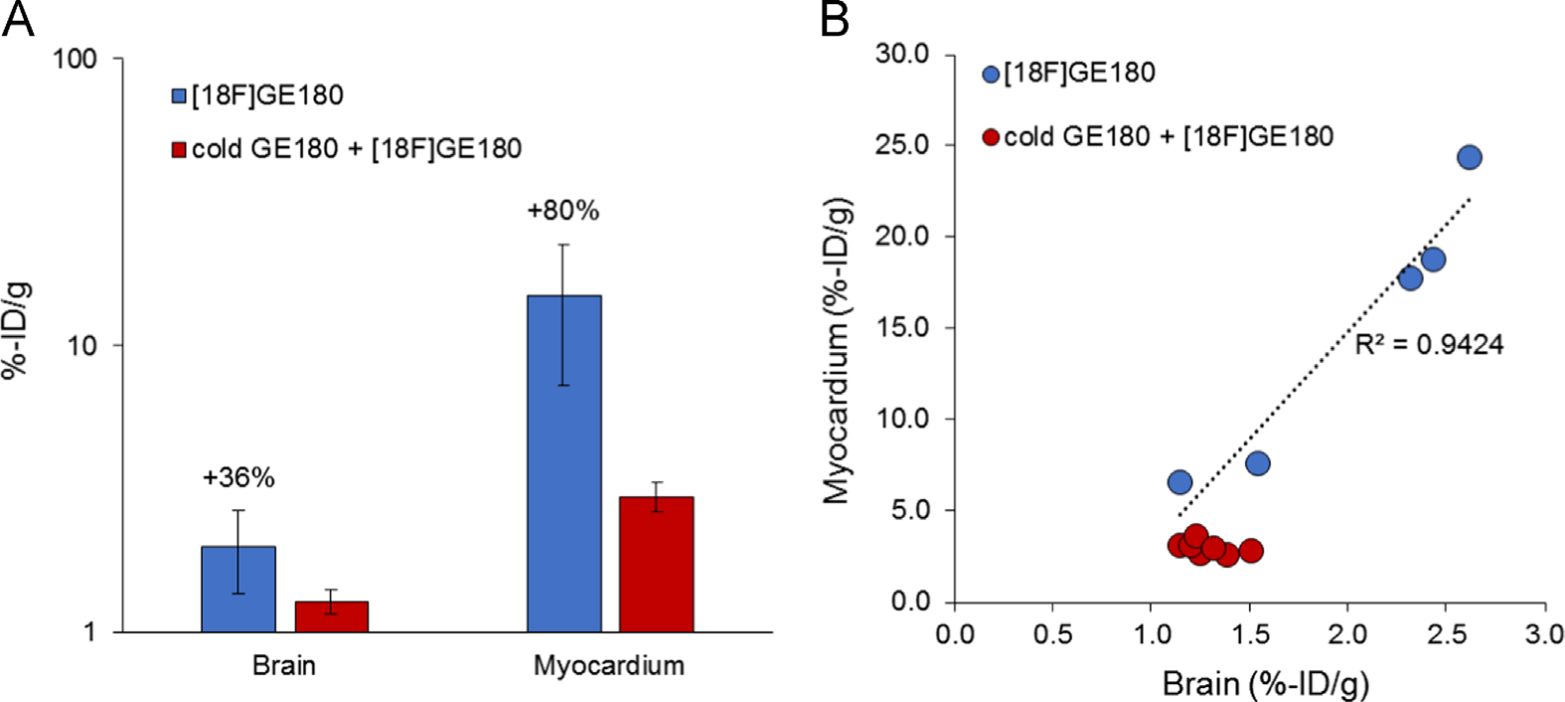
Gamma counter measurements of tracer uptake in brain and myocardium ex vivo*.* (A) Bar graphs (logarithmic scale) show %-injected dose-(ID)/g at 107 min after injection of [^18^F]GE180 with (red) and without (blue) prior blocking with excess non-radioactive GE180 (1000:1). Specific binding was estimated to be 36% in the brain and 80% in the myocardium. Absolute specific binding was 17-fold higher in the myocardium when compared to the brain. (B) Correlation between %-ID/g of brain and myocardium in single mice indicate a relationship between specific TSPO tracer binding in the two tissues, whereas nonspecific binding did not correlate (*R*^2^ < 0.2). Data derive from *N* = 5 unblocked and *N* = 7 blocked C57Bl/6 mice at seven months of age. Error bars indicate standard deviations. Single data points are available in the supplement.

**Fig. 2:**
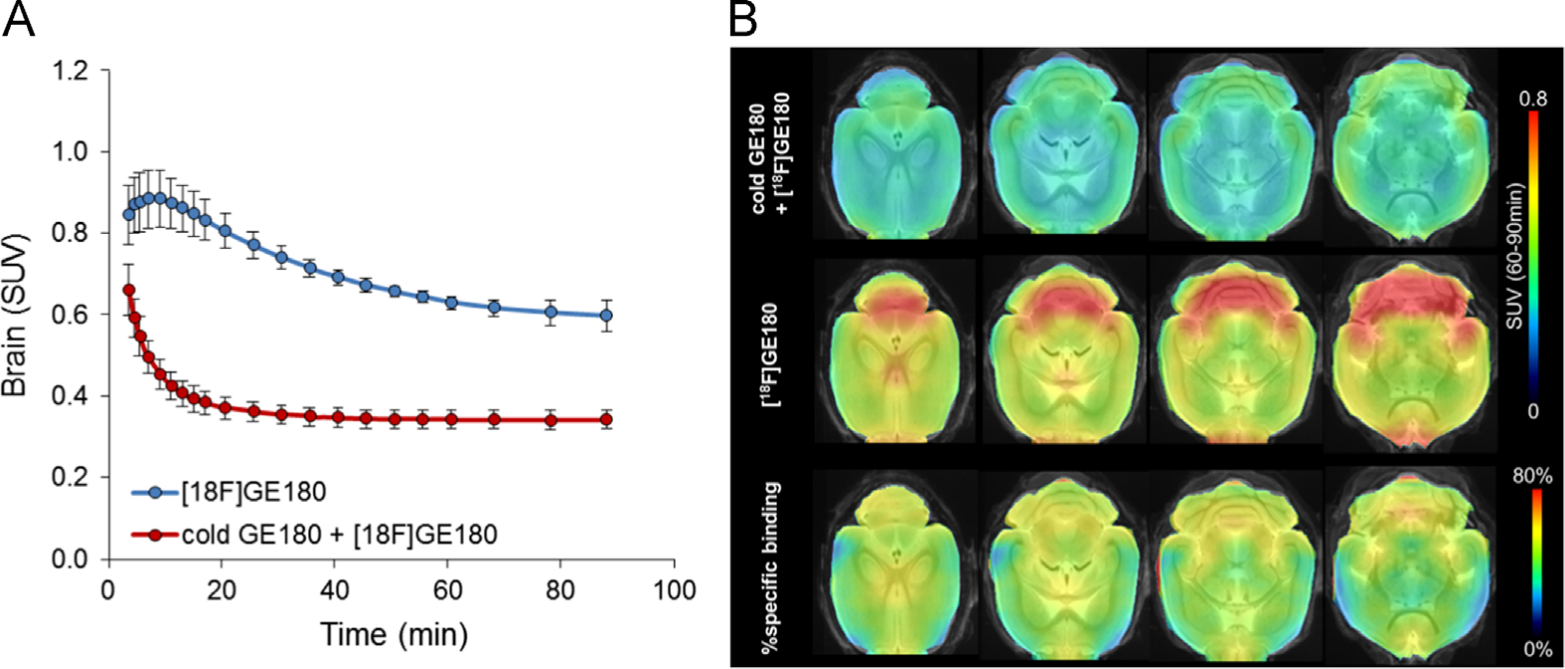
Dynamic and regional in vivo positron-emission-tomography (PET) measurements of the brain. (A) Mean dynamic brain PET standardized uptake value (SUV) plots for groups of unblocked (blue) and blocked (red) mice during 90 min after injection of [^18^F]GE180. Error bars indicate standard deviations. (B) Regional analysis shows axial slices of 60–90 min [^18^F]GE180 PET SUV projected upon a magnetic resonance imaging template [2]. Upper row illustrates mice with prior blocking by a large mass dose of GE180 (1000:1), whereas the middle row shows unblocked mice. Voxel-wise percentage of specific binding in the rodent brain is depicted in the bottom row. Highest specific binding was observed in regions with high abundance of ependymal glia cells, e.g. adjacent to the fourth ventricle, whereas specific binding in the cortex was low. Data derive from *N* = 3 unblocked and *N* = 5 blocked C57Bl/6 mice at seven months of age. Error bars indicate standard deviations. Single data points are available in the supplement.

**Fig. 3:**
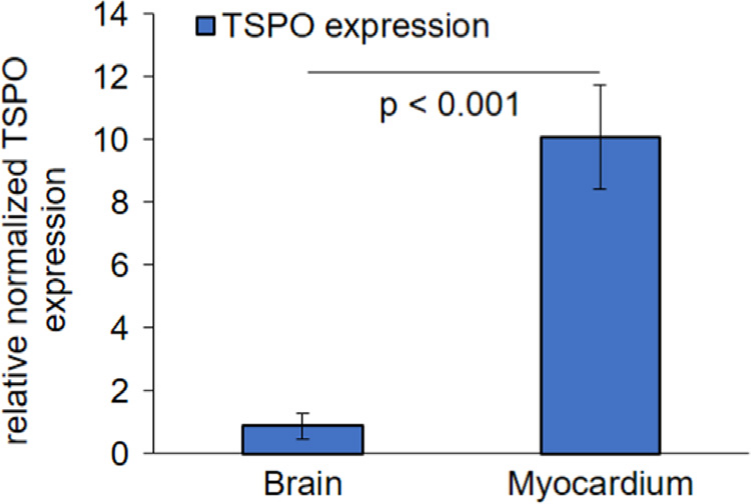
Quantitative polymerase chain reaction assessment of TSPO gene expression in brain and myocardium. Bar graphs show relative normalized (actin-beta and glyceraldehyde 3-phosphate dehydrogenase) TSPO expression as assessed by the quantitative polymerase chain reaction. TSPO expression was 11-fold higher in the myocardium compared to that in brain. Data derive from *N* = 6 C57Bl/6 mice at seven months of age. Error bars indicate standard deviations. Single data points are available in the supplement.

We observed that the myocardium presents a potential extra-cerebral reference region for [^18^F]GE180 TSPO positron-emission-tomography quantitation [1]. The following data provide additional information about the association and specificity of brain and myocardium TSPO tracer binding, as obtained by blocking experiments (Fig. 1, Fig. 2). Furthermore, we performed quantitative polymerase chain reaction for assessment of TSPO gene expression in brain and myocardium, independent of tracer-based measurements (Fig. 3).

## 2 Experimental design, materials and methods

### 2.1 Radiochemistry

Radiosynthesis of [^18^F]GE180 was performed as previously described [3], with slight modifications [4], in a procedure yielding radiochemical purity > 98%, and specific activity of 1400 ± 500 GBq/µmol at end of synthesis. For blocking, non-radioactive GE180 was used at a mass concentration ratio of 1000:1 compared to the radiolabeled ligand.

### 2.2 Gamma counter analysis

We performed blocking experiments with excess non-radioactive tracer for quantification of specific [^18^F]GE180 binding components in the rodent brain and myocardium. A total of twelve female C57Bl6 mice were analyzed at the age of seven months. Isoflurane anesthesia (1.5% in 2–4 l/min O_2_) was maintained during the experimental procedure, and mice were finally killed by cervical decapitation at 107 min post injection, while in a state of deep narcosis. Five mice received an injection of 14.7 ± 2.2 MBq [^18^F]GE180 into the tail vein (150 µl saline) Seven mice received an injection of excess non-radioactive GE180 (1000:1) prior to injection of 14.5 ± 2.1 MBq [^18^F]GE180 (in 150 µl saline) to a tail vein. Brain and myocardium tissues were quickly removed, and radioactivity concentration was measured in a gamma counter (Cobra Quantum 5002, Packard) with decay-correction to time of tracer injection [5].

### 2.3 Positron-emission-tomography acquisition and analysis

A subset of mice (*N* = 5 with blocking and *N* = 3 without blocking) was placed in the tomograph (Siemens Inveon DPET) immediately after injection of [^18^F]GE180, whereupon we began a 90 min dynamic emission recording as described previously [6]. A 15 min transmission scan was then obtained using a rotating [^57^Co] point source. The image reconstruction procedure consisted of three-dimensional ordered subset expectation maximization with four iterations and twelve subsets followed by a maximum a posteriori algorithm with 32 iterations. Framing was 3 × 60 s, 6 × 180 s, 9 × 300 s, 3 × 600 s. Scatter and attenuation correction were performed and a decay correction for [^18^F] was applied. With a zoom factor of 1.0 and a 128 × 128 × 159 matrix, a final voxel dimension of 0.78 × 0.78 × 0.80 mm was obtained.

Summations of the dynamic emission datasets were manually co-registered to a magnetic resonance imaging mouse atlas [2] by a rigid-body transformation using the PMOD fusion tool (V3.5, PMOD Technologies Ltd.), after blinding the reader to the mouse status (blocked/unblocked). In the second step, a reader-independent automatic re-registration to tracer-specific templates was performed, as reported previously [7]. Initial manual positron-emission-tomography to magnetic resonance imaging atlas fusion images were normalized by non-linear brain normalization to the template using the PMOD brain normalization tool (equal modality; smoothing by 0.6 mm; nonlinear warping; 16 iterations; frequency cutoff 3; regularization 1.0; no thresholding). The concatenation of both transformations was then applied to positron-emission-tomography frames in the native space, to obtain optimal resampling with a minimum of interpolation. The whole brain [^18^F]GE180 concentration (standardized uptake value; SUV) was measured in a volume-of-interest defined by the magnetic resonance imaging template. The volume-of-interest, comprising 525 mm^3^, included the entire cerebrum, cerebellum, brainstem and olfactory bulb. The 60–90 min image frames were used to calculate voxel-wise percentages of specific [^18^F]GE180 binding throughout the rodent brain.

## 3 Quantitative TSPO polymerase chain reaction

### 3.1 RNA isolation and reverse transcription

Brain and heart tissue from adult mice was immediately frozen to − 80 °C in liquid nitrogen after resection. Tissue was lysed with QIAzol Lysis Reagent (Quiagen) and homogenized. The InviTrap® Spin Universal RNA Mini Kit (Stratec) was used to isolate RNA and to separate it from contaminating DNA. RNA concentration was measured with NanoDrop™ 2000 Spectrophotometer (Thermo Fisher Scientific, Waltham, MA, USA). Extracted RNA was assessed based on spectral data and purity ratios (A260/A280). Reverse transcription from 50 µL of 50 ng/µL RNA was performed by incubating with 4 µL 25x dNTPs, 10 µL 10x random primers, 5 µL MultiScribe™ Reverse Transcriptase 50 U/µL, 21 µL Nuclease-free H_2_O for 10 min at 25 °C, then 120 min at 37 °C. The transcription was terminated by incubating at 85 °C for 5 min.

### 3.2 Real-time polymerase chain reaction

TSPO mRNA expression was measured using a CFX connect optics module RT-System (BioRad, Hercules, CA, USA). cDNA template 9 µl was added to 20× TaqMan® Gene Expression Assay Mm00437828_m1 1 µl and 2x TaqMan® Gene Expression Master Mix 10 µl. Thermal cycling conditions were 2 min at 50 °C and 10 min at 95 °C, followed by 40 cycles at 98 °C for 15 s and at 60 °C for 1 min. To test specificity of the polymerase chain reaction product a melt curve analysis was performed. TSPO expression was normalized to the housekeeping genes Actin-beta and glyceraldehyde 3-phosphate dehydrogenase expression was used for comparative quantification.
