# Supplementary material for: Data on specificity of [18F]GE180 uptake for TSPO expression in rodent brain and myocardium
Source: Data Brief. 2018 May 5;19:331–6. doi: 10.1016/j.dib.2018.04.133 (PMC5992977; doi:10.1016/j.dib.2018.04.133)
Supplement: Supplementary file 1 — Transparency document [file mmc1.docx]

The form is not available for download. Please send it via email
